# Bromide of Potassium in the Uncontrollable Vomiting of Pregnancy

**Published:** 1878-04-20

**Authors:** Samuel C. Busey

**Affiliations:** Washington, D. C., President of the Medical Society of the District of Columbia


### BROMIDE OF POTASSIUM IN THE UNCONTROLLABLE VOMITING OF PREGNANCY.

By Samuel C. Busey, M.D., Washington, D. C., President of the Medical Society of the District of Columbia.

As it is my purpose simply to set forth the utility of the potassium bromide in the treatment of this obstinate, but, fortunately, rare complication of pregnancy, I will not undertake any comparison of the relative merits of the numerous agents which have been recommended by their respective advocates. In the accomplishment of this object I am fortunate enough to possess the memoranda of two cases prepared by competent and impartial observers, to which cases I was called through their kindness and partiality. The first case occurred in the practice of my friend, Dr. P. J. Murphy.

Case I. "Mrs. McC., white, aged 28, a very stout and robust married lady, never had been pregnant, though married for five years, suffered a great deal at her menstrual periods from pain in the back and lower part of abdomen, flow always scanty, lasting usually but one day, very nervous and excitable. After treatment of several months the above symptoms were relieved, and she became pregnant in the spring of 1874. Two weeks subsequent to the date at which the menses should have occurred, profuse salivation ensued, several dozen handkerchiefs being required during the twenty-four hours; there was obstinate constipation, relieved temporarily by enemata, constant vomiting, the simplest nutriment being ejected. The various remedies recommended in such cases were tried, oxalate of cerium, minute doses of calomel, effervescing nitrate of cerium, iced champagne, etc., but to no purpose. My patient becoming alarmingly prostrated, I called to my assistance Dr. Busey, who ordered drachm doses of potass. bromide in two tablespoonfuls of beef-tea, injected per rectum, every four hours. This treatment completely arrested the vomiting in a few days, and with it all the nervous phenomena passed away. Mrs. McC. was now in the fifth month of pregnancy, and enjoyed comparatively good health until towards the close of the sixth month of pregnancy, when she aborted, the abortion arising partly from her debilitated condition and partly from undue excitement on hearing of sudden bad news."

To the above notes Dr. Murphy is kind enough to append the following history of the second pregnancy of the same lady:—

"In the summer of 1876 she again became pregnant, and the same phenomena which accompanied her first gestation were again observable. She was then under the care of an eminent physician in New Jersey, and her condition became such that I was telegraphed for, her friends supposing her to be in a dying condition.

"The physician in attendance had exhausted all his resources, and had called in another to assist him. When I arrived her condition was very critical, pulse small and thready, 120 per minute, great restlessness, skin hot, tongue dry, brown, and furred, great pain over epigastrium, and all the symptoms accompanying great exhaustion.

"I recommended the treatment pursued with such success in her former sickness, and left, asking to be informed of the result.

"Almost immediately after the third injection she was relieved. Mrs. McC. went to full term, and I had the pleasure of delivering her of a fine healthy female child."

The second case exhibits more markedly the salutary effects of the potassium bromide. It occurred in the practice of Dr. Mackall, whose extensive experience and accurate observation entitle his opinion to the highest consideration. The following extract from a letter from him furnishes the preliminary details of the case:—

CASE II. "Mrs. E.'s husband consulted me in my office about the 25th of April, 1877, with reference to her condition. He stated that his wife was three or three and a half months pregnant with her first child, and had been free from nausea and vomiting until a few days prior to his seeing me; but that these symptoms had suddenly become marked and distressing. As she declared that she would not see a physician, he thought it best that I should prescribe without visiting her. Accordingly I directed some simple medicine (I think, trisnit. of bismuth.) On the following day I learned that the medicine had afforded no relief. It was, therefore, discontinued, and oxalate of cerium substituted. This also failed, and, from the description of her condition, I felt that her wish not to see a physician should be longer regarded. On visiting her I found her very ill; pulse barely perceptible, extreme restlessness, extremities cold, vomiting incessantly. During the night and morning she had repeatedly ejected blood, and was still now and then vomiting it up in quantities of a tablespoonful or more. The tip and sides of the tongue were red and glazed. Great tenderness on pressure over the epigastrium. She had not slept, nor retained a particle of food for two days.

"During the next twenty-four hours, there was no recurrence of the hæmatemesis, but otherwise her condition remained unchanged, although every means that suggested itself was faithfully tried.

"Another day passed without improvement. No medicine, no form of nutriment could be retained by the stomach (her only support was by means of nutritive and stimulating enemata). A blister applied over the pit of her stomach, morphia injected hypodermically, starch, and laudanum enemata and other measures failed in accomplishing material benefit. A vaginal examination being made, marked anteflexion of the womb, with enlargement and tenderness of the fundus, was revealed. I now requested her friends, who had been previously apprised of her danger, to have you called in consultation, and you are familiar with its subsequent history. I will leave to you the further description of the case, together with the treatment adopted. In conclusion, however, I desire to express my conviction that the large enemata of bromide of potash, which you suggested, were mainly instrumental in relieving the gastric irritability. I would also state that when the nausea and vomiting were arrested, they ceased, I may say, abruptly, and did not again recur, except for a few hours once or twice several weeks after her convalesence.

"Further, to show to what extent the patient bad been reduced, I mention the fact that she could not be even raised up in bed for about four weeks, and six weeks or more elapsed before she could be lifted into a chair for a few moments.

"I saw Mrs. E. this morning; she is now perfectly well, and for several months has been free from any unpleasant symptoms."

In addition to the symptoms enumerated by Dr. Mackall, there were present at the time of my first visit great tremulousness followed by sinking, which came on in paroxsms, usually occurring when any person unexpectedly approached her bedside, or when any effort to move was made. The pulse was barely, if at all, perceptible, the surface was cold, capillary circulation very languid, voice very feeble, and, when any attempt was made to speak, retching immediately ensued. Her expression was anxious and distressed. Forty grains of the potassium bromide dissolved in a mixture of beef-tea and a half ounce of brandy, to which were added ten drops of laudanum, were ordered to be administered per annum every four hours. The stomach to remain at rest, nothing whatever to be given per orem until further orders. The beneficial effects were manifest after the third enema, and, when 480 grains of the bromide had been administered, the nausea and vomiting had entirely ceased. After the first twenty-four hours the interval between the enemata was lengthened, and she was allowed to take nutriment in very small quantities, at short intervals, by the mouth. Even after the discontinuance of the nausea and vomiting, and the suspension of the bromide and nutritive enemata, the alarming prostration was so persistent, notwithstanding the ingestion of what seemed to be an adequate amount of nutriment and stimulants by the mouth, that the propriety of induction of abortion was entertained and discussed. During this period, which lasted several days, the brain seemed overwhelmed by the exhaustion, even though the heart had regained in a measure force and rhythm. Happily, however, interference was delayed, the expectant plan of treatment persisted in, and complete reaction ensued. As stated by Dr. M., she is now well, and expects to be confined during the ensuing month of November, 1877.

As a rule, the bromide, in doses varying from 30 grains to one drachm, dissolved in beef-tea, to which brandy and laudanum may or may not be added, should be given every four hours until the nausea and vomiting have ceased, and the stomach will retain some bland food, and stimulants if necessary, and then it should be gradually withdrawn by extending the intervals between the enemata. This treatment has not failed in any case which has come under my observation; but the practitioner must not imagine that with the suspension of the nausea and vomiting the case is concluded. The effects of the deprivation of food and fluids, together with the nervous and circulatory disturbances, may seriously protract convalesence, and excite the gravest apprehensions.

In conclusion, I must add that the method of treatment is not original with me. To Dr. Girabetti is due the credit of having first suggested and successfully applied this mode of administering the potassium bromide in obstinate vomiting of pregnancy. He administered it in increasing doses, giving 92 grains the first day, 8 grammes the second, and 10 the third; after which he lessened the dose in proportion to the effect produced.—*Amer. Jour. Med. Sciences.*

